# Polyploidy on Islands: Its Emergence and Importance for Diversification

**DOI:** 10.3389/fpls.2021.637214

**Published:** 2021-03-04

**Authors:** Heidi M. Meudt, Dirk C. Albach, Andrew J. Tanentzap, Javier Igea, Sophie C. Newmarch, Angela J. Brandt, William G. Lee, Jennifer A. Tate

**Affiliations:** ^1^Museum of New Zealand Te Papa Tongarewa, Wellington, New Zealand; ^2^Institute of Biology and Environmental Sciences, University of Oldenburg, Oldenburg, Germany; ^3^Ecosystems and Global Change Group, Department of Plant Sciences, University of Cambridge, Cambridge, United Kingdom; ^4^School of Fundamental Sciences, Massey University, Palmerston North, New Zealand; ^5^Manaaki Whenua – Landcare Research, Dunedin, New Zealand

**Keywords:** colonization, diversification, endemism, island floras, ploidy level, phylogenetic path analysis, polyploidy, whole genome duplication

## Abstract

Whole genome duplication or polyploidy is widespread among floras globally, but traditionally has been thought to have played a minor role in the evolution of island biodiversity, based on the low proportion of polyploid taxa present. We investigate five island systems (Juan Fernández, Galápagos, Canary Islands, Hawaiian Islands, and New Zealand) to test whether polyploidy (i) enhances or hinders diversification on islands and (ii) is an intrinsic feature of a lineage or an attribute that emerges in island environments. These island systems are diverse in their origins, geographic and latitudinal distributions, levels of plant species endemism (37% in the Galapagos to 88% in the Hawaiian Islands), and ploidy levels, and taken together are representative of islands more generally. We compiled data for vascular plants and summarized information for each genus on each island system, including the total number of species (native and endemic), generic endemicity, chromosome numbers, genome size, and ploidy levels. Dated phylogenies were used to infer lineage age, number of colonization events, and change in ploidy level relative to the non-island sister lineage. Using phylogenetic path analysis, we then tested how the diversification of endemic lineages varied with the direct and indirect effects of polyploidy (presence of polyploidy, time on island, polyploidization near colonization, colonizer pool size) and other lineage traits not associated with polyploidy (time on island, colonizer pool size, repeat colonization). Diploid and tetraploid were the most common ploidy levels across all islands, with the highest ploidy levels (>8*x*) recorded for the Canary Islands (12*x*) and New Zealand (20*x*). Overall, we found that endemic diversification of our focal island floras was shaped by polyploidy in many cases and certainly others still to be detected considering the lack of data in many lineages. Polyploid speciation on the islands was enhanced by a larger source of potential congeneric colonists and a change in ploidy level compared to overseas sister taxa.

## Introduction

Since the time of Darwin (1839), the study of island biotas has provided major advances that have profoundly influenced ecological, evolutionary, and biogeographical theory (MacArthur and Wilson, 1967; Mayr, 1967; Carlquist, 1974; Emerson, 2002). Island biotas are generally the net outcome of immigration (dispersal and establishment), local diversification, and extinction (Carlquist, 1974), and these processes are known to be influenced by specifics of the island, such as age, area, distance from nearest potential source floras (Rosenzweig, 1995), and local habitat (abiotic and biotic) conditions (Savolainen et al., 2006; Vamosi et al., 2018). Islands may also pose selective filters that may be apparent in the intrinsic traits of species, including those increasing their ability to disperse, colonize, and establish in novel habitats (Crawford et al., 2009; Vargas et al., 2015). Among those traits, whole genome duplication or polyploidy has been suggested to be central to facilitating long-distance dispersal (Linder and Barker, 2014), the survival of small populations (Rodriguez, 1996), and evolution of novel traits (Soltis et al., 2014), all features of many island floras. Polyploids are broadly categorized as autopolyploids, when formed within a species, or allopolyploids, when formed between genetically distinct species (Stebbins, 1947). Polyploidy, especially allopolyploidy, may also be a mechanism for increasing the genetic diversity of the colonizer (Crawford and Stuessy, 1997; Carr, 1998), which is often low for island colonizers and lineages (e.g., Frankham, 1997; but see García-Verdugo et al., 2015). Thus, polyploidy could have multiple advantages, particularly in island floras.

**Figure 1:**
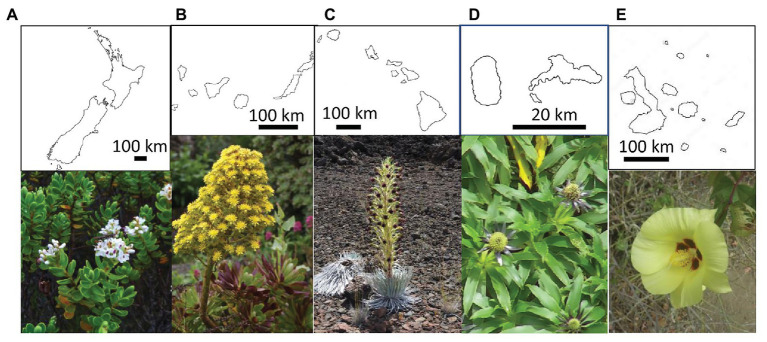
Outline and representative polyploid plants from the five analyzed island systems. From left to right: **(A)** New Zealand: *Veronica topiaria* (Plantaginaceae) © Phil Garnock-Jones; **(B)** Canary Islands: *Aeonium arboreum* (Crassulaceae) © Dirk Albach; **(C)** Hawaiian Islands: *Argyroxiphium sandwicense* (Asteraceae) © Marc Appelhans; **(D)** Juan Fernández Islands: *Eryngium bupleuroides* (Apiaceae) © Lukas Mekis; **(E)** Galápagos Islands: *Gossypium darwinii* (Malvaceae) © A. Emmerson.

Despite its potential benefits, polyploidy has been historically suggested to play a minor role in diversification of island floras, with many groups showing “chromosomal stasis” (Carr, 1998; Stuessy and Crawford, 1998). For the oceanic island systems that inform this perspective (Hawaiian, Juan Fernández, Galápagos, and Bonin Islands), paleopolyploidy was suggested to have helped some lineages establish, with little polyploidization thereafter (Carr, 1998; Stuessy and Crawford, 1998), such as in *Gossypium* (Malvaceae; Figure 1). Thus, while polyploid, these oceanic island lineages remained chromosomally static. By contrast, chromosomal variation was found in two island systems near their continental source, the Queen Charlotte Islands and Canary Islands (Stuessy and Crawford, 1998). Although Crawford et al. (2009) began to address the question of the evolution of polyploidy in island systems and its role in diversification (for Asteraceae; Crawford et al., 2009), others discounted its impact (Stuessy et al., 2014; Crawford and Archibald, 2017). However, in those studies (Carr, 1998; Stuessy and Crawford, 1998; Crawford et al., 2009), chromosome counts from only a small percentage of native island species were available, so interpretations were based on island origin (continental vs. oceanic) and island age rather than polyploidy itself. Other island systems, in particular New Zealand, have not been included in these larger comparative studies, despite chromosome numbers being widely available for many native plant species (see Dawson, 2000, 2008). Furthermore, a phylogenetic context was lacking in previous studies, which is important because the phylogeny will indicate the number of origins on the island as well as patterns of diversification on the island related to polyploidy or other factors.

As robust (and increasingly, dated) phylogenetic hypotheses have amassed over the last several decades, along with continued efforts to document chromosome numbers, we now have the capability to test the role of polyploidy in contributing to species diversification on islands in a phylogenetic context (Kellogg, 2016; Crawford and Archibald, 2017). As species in many different island floras are known to have diverse chromosome numbers and form species-rich groups, they may not be chromosomally static lineages as previously thought (Soltis et al., 2009; New Zealand: Murray and de Lange, 2011; Canary Islands: Caujapé-Castells et al., 2017). A recent analysis of the global distribution of polyploids indicated polyploid frequency is highest in temperate areas rich in perennial herbs, including mountainous areas (Rice et al., 2019). Analyzed from a global perspective, several island systems are polyploid-rich, including the Hawaiian Islands (50% of analyzed species are polyploid), New Zealand (46%), and Galápagos Islands (46%), whereas others have lower polyploid frequencies – such as the Canary Islands (32%) – or insufficient data (Juan Fernández: 0 of 2 species included were polyploid; Rice et al., 2019). However, that study was focused primarily on assessing external drivers of polyploid plant distribution globally, not on island systems, and did not utilize dated phylogenies. A dated phylogenetic context allows a more precise determination of when diversification and polyploidization have occurred within each lineage (i.e., as separate events), and puts the focus on (multiple) lineage ages rather than a single island age. Such an approach can generate multiple independent data points that can be analyzed together to address the timing and role of polyploidy on islands. Dated phylogenies are especially important in the context of island diversification because they make it possible to estimate *in situ* diversification rates and to consider in the analyses the geological processes (e.g., volcano eruptions, inundations) that may affect lineages at different evolutionary time scales.

**Figure 2:**
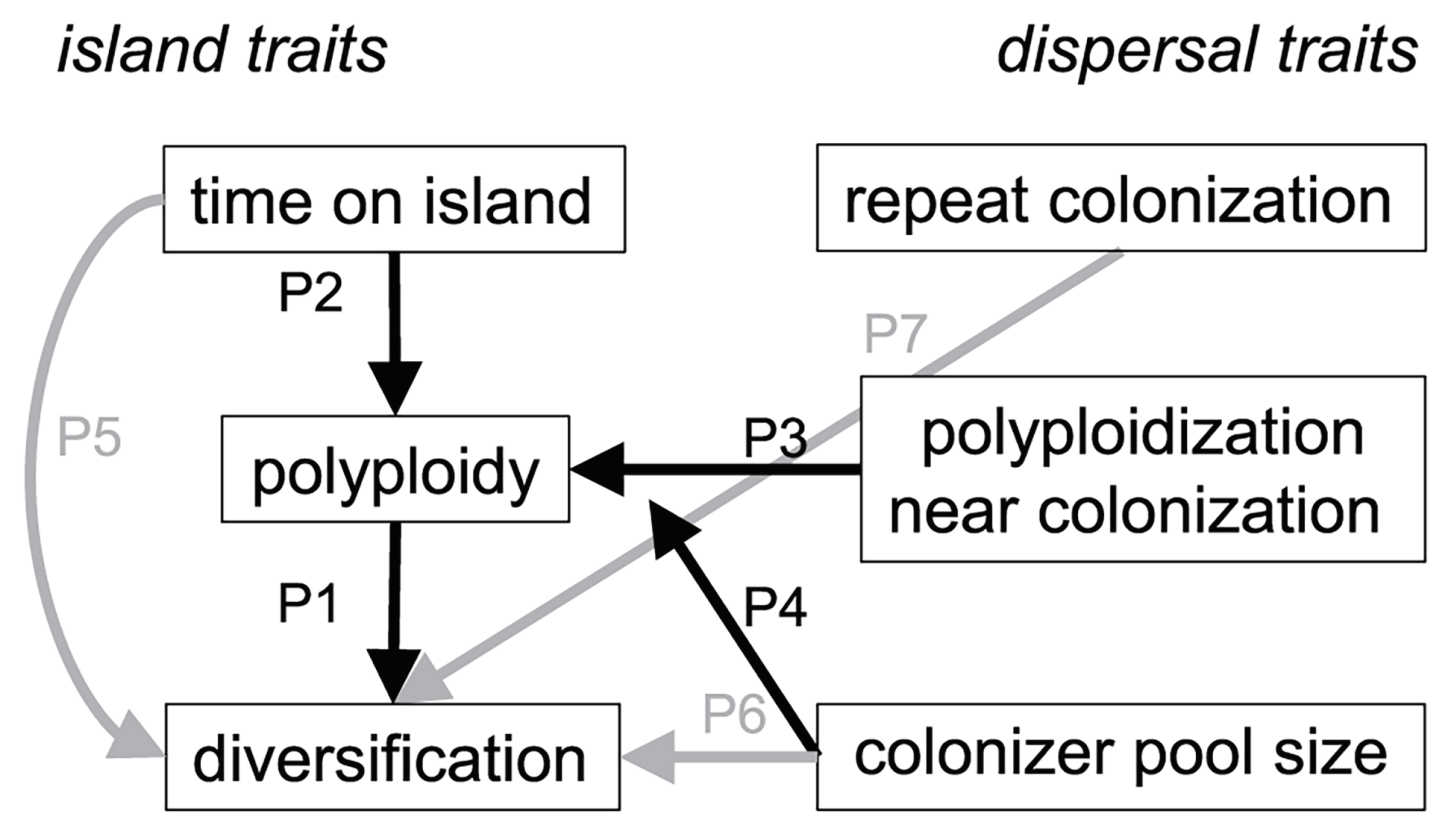
Conceptual model for how species diversity in island lineages varies with polyploidy and extrinsic colonization history. Arrows show predicted linkages between direct and indirect effects of polyploidy (P1–P4, shown in black) and non-polyploidy effects (P5–P7, shown in gray) on diversification of island floras. Left-hand of the plot shows traits intrinsic to island lineages whereas the right-hand shows traits associated with island dispersal and colonization.

We hypothesize that polyploidy has played an important role in the diversification of island floras by facilitating dispersal and establishment of plants to islands, and/or by generating additional diversity through varying ploidy levels. To test a conceptual model for how polyploidy influences species diversification on islands, we synthesized published chromosome and divergence time data for 150 lineages representing 1,805 endemic species across the Juan Fernández, Canary, Hawaiian, and New Zealand archipelagos (Figure 1). All these island systems, except New Zealand, have been included in previous comparative studies or reviews of polyploidy and diversification (see above), but without the time-calibrated phylogenetic context we add here. By using phylogenetic path analysis, we simultaneously tested the strength and direction of causal associations to explain the diversity of endemic island species in these four archipelagos. We predicted that island lineages would be more diverse (i.e., have more endemic species) if they (Figure 2): (P1) contained multiple ploidy levels; (P2) had more time to generate ploidy levels once on these islands, as indicated by the length of time they were present on an island; (P3) had a different (i.e., higher) ploidy level relative to their sister lineage, indicating polyploidization immediately before or after colonization of the island; (P4) were derived from a large pool of overseas congeners and thus the likelihood of polyploidization events that could enhance diversity was greater. We also tested other direct explanations that did not involve ploidy and predicted island lineages would be more diverse if they (Figure 2); (P5) were older, because they have simply had more time to undergo speciation and isolate populations across a greater availability of niches (e.g., Lee et al., 2012; Tanentzap et al., 2015); (P6) were derived from a large pool of overseas congeners and thus the probability of colonization from that pool would be higher; and (P7) repeatedly colonized islands with different ancestral species, indicated by a lack of monophyly. Hypotheses P1–P4 test effects of ploidy that are both direct (P1) or indirect (P2–P4) and mediated by time (P2), changes to the ploidy levels themselves (P3), or source pool size (P4). Alternatively, hypotheses P1, P2, and P5 test polyploidy as a trait important in diversification, whereas P3, P4, P6, and P7 test polyploidy as a trait important in dispersal or establishment.

## Materials and Methods

### Data Collection

We compiled data of indigenous species, subspecies, varieties, and forms (hereafter: species), and noted whether they were native or endemic, for vascular plants on five island systems using published references for the Canary Islands (CI; Arechavaleta et al., 2010), Galápagos Islands (GI: Jaramillo Díaz et al., 2017, 2018), Hawaiian Islands (HI: Wagner et al., 2005 and electronic updates; Imada, 2012), Juan Fernández Islands (JF: Table 5.1 in Stuessy et al., 2017), and New Zealand (NZ: Breitwieser et al., 2012; Schönberger et al., 2019). We then added data for each species (where known and when from a sample from that island system) on chromosome number (Index to Plant Chromosome Numbers,^1^ Chromosome Counts Database,^2^ and additional literature search), genome size (Pellicer and Leitch, 2020 ^3^) and ploidy level. We summarized this information for each genus on each island system, including the total number of indigenous and endemic species, whether the genus was endemic to the island system, chromosome number, genome size, and ploidy level (Table 1). For each genus with at least one native species on a particular island group, we identified the most recent phylogenetic study (that included at least one native species sampled from that island) to determine if the genus was monophyletic on the island system and whether there was a change in ploidy level compared to the sister group. For lineages with dated phylogenies, we extracted the mean time of their divergence from both sister lineages and their most recent common ancestor (i.e., stem and crown ages, respectively). Values were either given in papers or estimated using WebPlotDigitizer^4^ on dated tree figures. We also estimated the variance for each crown and stem age using the standard deviation (SD) or assuming normality of the longer of the two tails of the 95% highest posterior density or confidence intervals (after Lee et al., 2012; Tanentzap et al., 2015; Brandt et al., 2016). For genera that were not monophyletic within the archipelago, we used dates of the earliest-derived species or lineage on the island system. From these data (see Supplementary Files), we calculated several comparative statistics (Table 2), as described below.

**Table 1:** Summary of island systems for all genera and species^1^ of native vascular plants.

	Archipelago				
Variable	New Zealand^a^	Canary Islands^b^	Hawaiian Islands^c^	Juan Fernández^d^	Galápagos^e^
No. of islands	3 main islands plus ~600 smaller islands/islets	7 main islands plus 6 smaller islands/islets	8 main islands, plus 129 smaller islands/islets	3 islands	18 main islands, plus 110 smaller islands/islets/rocks
Area of islands	268,021km^2^	7,493km^2^	28,311km^2^	100.2km^2^	7,985km^2^
Shortest distance from nearest continental neighbor	1,490km E of Australia	96kmW of Morocco	3,500km SW of United States	580kmW of Chile	930kmW of mainland Ecuador
Main reference for data in rows below	Schönberger et al. (2019) ^2^	Arechavaleta et al. (2010)	Wagner et al. (2005 and electronic updates); Imada (2012)	Stuessy et al. (2017)	Jaramillo Díaz et al. (2017, 2018)
No. native species	2,551	1,677^3^	1,233	209	550
No. endemic species (%)	2,096 (82%)	647 (39%)	1,082 (88%)	136 (65%)	204 (37%)
No. species with chromosome numbers (%)	1,962 (77%)	1,171 (70%)	414 (34%)	87 (42%)	39 (7%)
No. species with genome size estimates (%)	245 (12.5%)	237 (14%)	29 (2%)	0	0
No. native genera	430	466	272	103	277
No. endemic genera (%)	48 (11%)^4^	22 (5%)	30 (11%)	12 (11%)	7 (3%)
No. genera with multiple ploidies on island (% of total/% of genera with at least two native species)	88 (21%/35%)	96 (21%/40%)	27 (10%/19%)	5 (5%/13%)	3 (1%/3%)
Range of ploidy levels in one genus	1–6	1–4	1–2	1–2	1–2
Most common ploidy levels (range)	2*x*, 4*x*, 6*x* (2*x*–20*x*)	2*x*, 4*x* (2*x*–12*x*)	2*x*, 4*x* (4*x*–6*x*)	2*x*, 4*x* (2*x*–8*x*)	2*x*, 4*x* (2*x*–4*x*)
No. genera with two or more native species on island (%)	256 (60%)	239 (51%)	139 (51%)	39 (38%)	105 (38%)
No. genera^5^ for which a molecular phylogeny is available (% of native genera)	205 (48%)	170 (36%)	86 (32%)	31 (30%)	71 (26%)
No. genera^5^ for which a dated molecular phylogeny is available (% of native genera)	112 (26%)	41 (9%)	27 (10%)	8 (8%)	4 (1%)
No. total genera (%) / no. genera^5^ (%) for which there is at least one known chromosome number^6^	389 (92%)/250 (98%)	406 (87%)/219 (92%)	155 (57%)/92 (67%)	58 (56%)/25 (64%)	21 (8%)/13 (12%)
No. species per genus range/average/median	1–143/6.0/2	1–33/3.6/2	1–80/4.5/2	1–12/2.0/1	1–19/2.0/1
No. gymnosperm species (% endemic)	22 (100%)	6 (33%)	0	0	0
No. gymnosperm genera (% endemic)	10 (30%)	3 (0%)	0	0	0
No. fern species (% endemic)	210 (46%)	50 (6%)	167 (75%)	57 (46%)	125 (9%)
No. fern genera (% endemic)	57 (4%)	23 (0%)	51 (4%)	24 (2%)	52 (0%)

*Island geographic data from:*

a *McGlone et al. (*2001).

b *Carracedo and Troll (*2016).

c *Price (*2004).

d *Stuessy et al. (*1998).

e *Rivas-Torres et al. (*2018).

1 *Includes species, subspecies, varieties, and forms; collectively called “species” for simplicity*.

2 *Most data generally agree with* Breitwieser et al. (2012).

3 *The species numbers here include 288 subspecies, which means they will be higher than in* Arechavaleta et al. (2010) *which only counts species*.

4 *But see* Garnock-Jones (2014) *who suggested the actual number of endemic genera is lower, ca. 28–44 (7–10%)*.

5 *With two or more native species on the island*.

6 *From a specimen of at least one native species from the island*.

**Table 2:** Summary of variables used for the statistical analyses.

	Island archipelago				
Variable	New Zealand	Canary Islands	Hawaiian Islands	Juan Fernández	All islands
No. of lineages	98	23	23	6	150
Number of monophyletic island lineages	33	13	18	3	67
Number of lineages with different ploidy level than closest sister outside of island	8	2	6	2	18
Mean (SD) number of island endemic species per lineage	11.3 (17.6)	13.7 (15.0)	16.4 (19.0)	4.5 (3.7)	12.2 (17.1)
Mean (SD) stem age per lineage in millions of years	15.63 (17.30)	8.48 (6.19)	9.21 (6.23)	5.93 (5.86)	13.16 (14.81)
Mean (SD) number of ploidy levels per lineage	1.7 (1.2)	1.5 (0.7)	1.2 (0.4)	1.0 (0.0)	1.6 (1.1)
Mean (SD) % of endemic island species with chromosome counts	86.0 (22.8)	74.3 (24.0)	50.9 (31.9)	71.8 (36.1)	78.2 (27.8)
Median number of species in lineage outside of island system	34	77	58	384	51

Means and standard deviations (SD) were calculated for each non-count variable. The data set included the subset of 150 lineages of native vascular plants with the following criteria: dated phylogeny available, at least one native species sampled in the phylogeny, at least two native species on the island system, and at least one known chromosome count from the island system.

### Statistical Analyses

We tested our conceptual model (i.e., P1–P7) by fitting two separate phylogenetic least squares (PGLS) regression models using the *gls* function (implemented in the nlme package; Pinheiro et al., 2014) in R v.3.6, which were united into a single path analysis with the *piecewiseSEM* package (Lefcheck, 2016). As we were interested in the effect of ploidy on diversification, we analyzed lineages that had at least two native species on the island system, a dated phylogeny available with at least one native island species, and at least one chromosome count of a native island specimen (Supplementary File 1). Lineages that fit our criteria were included from New Zealand (*n* = 98), Canary Islands (*n* = 23), Hawaiian Islands (*n* = 23), and the Juan Fernández Islands (*n* = 6), but data from Galápagos Islands were insufficient for downstream analysis. These lineages were always genera aside from four exceptions that were closely related genera that formed a monophyletic group on the archipelago (Supplementary File 1). The models were fitted to the number of endemic species and number of ploidy levels for each lineage on each island system. To predict species number, we modeled observations based on both log-transformed stem age and number of ploidy levels of the island lineage, total number of accepted and unresolved species in the lineage outside of the island system according to The Plant List v1.1 (2013), and separate binary variables for whether the lineage was monophyletic on the island system and its ploidy level was different from its closest sister lineage outside of the island system. We let the effects of ploidy levels vary among the four island systems and accounted for differences in the mean endemic diversity of lineages among island systems. Doing so also ensured that island systems with more data points, such as New Zealand, did not bias the estimated effects towards themselves. We used stem ages to represent lineage age because they were better sampled than crown ages (*n* = 150 vs. 116 lineages, respectively), were highly correlated with crown age (Pearson correlation between mean ages, *r* = 0.84, *df* = 110, *p* < 0.001), and may better reflect the entire evolutionary history of clades (Scholl and Wiens, 2016). The model of archipelago ploidy levels was identical to that for endemic diversity except without including ploidy levels and archipelago monophyly as predictors and letting the stem age effect vary with the island system. Following standard practice, the models were fitted assuming the expected covariance in the responses between any two lineages was proportional to their shared evolutionary history along a phylogenetic tree, i.e., a Brownian motion (Symonds and Blomberg, 2014). Distances were derived by pruning the largest time-calibrated phylogenetic tree available for vascular plants, which contained 74,531 species and was generated with hierarchical clustering analysis of GenBank data and a backbone provided by Open Tree of Life version 9.1 (Smith and Brown, 2018; Jin and Qian, 2019). On average, sister branches in this phylogeny had an overlap of 1,792 base pairs, which corresponds to roughly one or two gene regions. Although the responses were counts and so could also be modeled with other approaches (e.g., phylogenetic generalized linear models), log-normal transformations made the responses normally distributed, as expected for some Poisson distributed variables. The *gls* function also had the advantage that it could be used to fit a path analysis and incorporate uncertainty in the responses unlike these other regression approaches. We specifically accounted for uncertainty in divergence time estimates and different levels of data completeness by weighting observations of species counts and ploidy levels with the inverse square root of divergence time SDs and the proportion of species with chromosome counts, respectively, after Garamszegi and Møller (2010).

## Results

### Comparison of Island Groups

The five island systems (New Zealand, Canary Islands, Hawaiian Islands, Juan Fernández, and Galápagos Islands) differ regarding number and total area of island system, distance to nearest continent, number (and percentage) of native and endemic species and genera, and data availability for phylogenies, dated phylogenies, chromosome numbers, genome size, and ploidy information (Table 1). The island systems have a five-fold difference in the number of indigenous genera (103 in JF to 466 in CI), a 10-fold difference in the number of native species (209 in JF to 2,551 in NZ), and a 20-fold difference in the number of endemic species (136 in JF to 2,056 in NZ). For simplicity, we chose to lump sub-specific ranks under species, so our determination of absolute species numbers may differ from other sources and we acknowledge that there is much taxonomic uncertainty in island radiations. We do not think that this approach has affected our analyses other than minor differences in species numbers.

On all island systems, the majority of indigenous vascular plant species are angiosperms, with a small percentage of ferns and even fewer gymnosperms. The average number of native species per genus is 2.0 (JF, GI), 3.6 (CI), 4.5 (HI), and 6.0 (NZ). The percentage of endemic genera was low for all island systems (3–11%), as was the availability of genome size data (up to 14% in CI). The availability of chromosome numbers was highest for CI and NZ (70–77%) and lowest for GI (7%), with HI (34%) and JF (42%) also having relatively few counts. The number of native non-monotypic genera on each island system ranged from 39 (38%, JF) to 256 (60%, NZ). On all island systems, molecular phylogenies have been published for the majority of these non-monotypic genera, ranging from 62% in HI to 80% in NZ, but dated phylogenies are less prevalent (4% in GI to 44% in NZ).

Of all the island systems studied here, New Zealand has the largest area, the lowest number of main islands (but the highest number of total islands including smaller islands), the largest flora, and the highest percentage of data available for its species (Table 1). Ferns comprise about 9% of species (210 species, 46% endemic) and 13% of genera in the native plant vascular flora [57 genera, of which only monotypic *Leptolepia* (Dennstaedtiaceae) and *Loxsoma* (Loxsomaceae) are endemic]. With respect to gymnosperms, all 22 species and one-third of the 10 native genera are endemic. Of the 430 NZ vascular plant genera, 256 have at least two native species, and the rest are monotypic on the archipelago. Of these 256, 78% have a phylogeny but only 46% have dated phylogenies. About 70% of genera with a phylogeny (144 genera) have 50% or more species included in phylogenies. Roughly one-third of these genera are monophyletic or nearly so in NZ (i.e., one NZ origin likely), one-third are not monophyletic in NZ (i.e., more than one origin in NZ), and about 40% are unknown due to lack of phylogeny or sampling. Ninety percent of the genera have at least one species with a chromosome count, but there are few published genome size estimates (12.5%, see Table 1). Fifty-seven genera have 10 or more native species, and two of these have over 100 species, i.e., *Veronica* (Plantaginaceae, 143 species, 96% endemic) and *Carex* (Cyperaceae, 118 species, 88% endemic). Ninety-four percent of genera with phylogenies have at least 50% of the species with chromosome counts. Genera have between 1 and 6 ploidy levels represented on the archipelago, ranging from 2*x* to 20*x*. Of the 199 NZ genera with phylogenies, 40% have multiple ploidies (the majority with two or three ploidy levels).

The Canary Islands rank second after NZ in terms of number of native species and data availability, third in terms of area, and first in terms of proximity to the nearest mainland (Table 1). They have the second-lowest percentage of endemic species (39%). There are 27 genera with 10 or more species, including several adaptive radiations, two of which comprise larger lineages of multiple genera, i.e., the *Aeonium* alliance (Crassulaceae; 62 endemic species in four genera) and the *Sonchus* alliance (Asteraceae; 62 endemic species in six genera; Kim et al., 2008). Gymnosperms are rare in CI (only six native species, two of which are endemic), and of the 50 fern species, only 6% are endemic; there are no endemic genera of gymnosperms or ferns. Of the 466 genera, 217 have between 2 and 33 species, and the rest are monotypic on the archipelago. Of these 217, 78% have a molecular phylogeny but only one-third are dated phylogenies. Between 1 and 27 native CI species per genus (17–100%) are included in the phylogenies; over half have 50% or more species included in phylogenies. Roughly 20% of these genera with phylogenies are monophyletic or nearly so in CI (i.e., one CI origin likely), 30% are not monophyletic in CI (i.e., more than one origin in CI), and about half are unknown due to lack of phylogeny or sampling. Eighty-seven percent of the genera have at least one species with a chromosome count, and there are genome size estimates known for about 14% of species. Ninety-four percent of genera with phylogenies have at least 50% of the species with chromosome counts. Genera have between 1 and 4 ploidy levels represented on the islands, ranging from 2*x* to 12*x*, with the most common ploidies being diploid and tetraploid. Of the 170 genera with phylogenies, 40% have multiple ploidies (the majority with two ploidy levels).

The Hawaiian Islands have the second-largest land mass (after New Zealand) of the five island systems and the highest level of species endemicity (88%; Table 1). There are 272 native vascular genera, 139 (51%) have between 2 and 80 species, and the remainder are represented by one species on the archipelago. Ferns represent 14% of the vascular plant species diversity and show a high level of endemicity at the species level (75%). There are no native gymnosperm taxa in HI. Phylogenies are available for about one-third of the genera, and of these, one-third are dated. Overall, we identified 142 phylogenies that included Hawaiian taxa, and 39% of these were for groups in which only one species occurs in HI. The flora is not well characterized chromosomally at the species level overall (34% of species with known chromosome numbers), but there is broad representation at the generic level, with 57% of genera having at least one species known. Ploidy levels range primarily from diploid to octoploid, with diploid and tetraploid representing the majority of known levels. Overall, polyploid series with multiple ploidy levels are lacking but there are true dysploid series (e.g., *x* = 13, 14) that occur in several genera of the Hawaiian silversword alliance (Asteraceae, Carr, 1998). The most common scenario for HI taxa (with known chromosome numbers) is that these lineages are tetraploid relative to their overseas congeners and these show chromosomal stasis on the island system [e.g., angiosperms: the six genera of Campanulaceae, *Bidens* (Asteraceae), *Stenogyne* (Lamiaceae); ferns: *Deparia* (Athyriaceae)]. Among angiosperm genera with two or more species, 60 (43%) are the result of a single colonization event and 11 are considered to have diversified *in situ*. Representative adaptive radiations include the genera of the Lobelioideae (Campanulaceae), those in the Lamioideae (Lamiaceae), and the aforementioned silversword alliance (Asteraceae; Price and Wagner, 2018). Twenty-seven genera are the result of two colonization events and 10 have three or more introductions to HI.

The Juan Fernández Islands have the smallest flora and the smallest area, have the same low number of only three main islands as New Zealand, and are probably second to last in terms of data availability (Table 1). Of the 103 JF genera, 39 have between 2 and 12 species, and the rest are monotypic on the archipelago. Only three genera have more than 10 native species, i.e., *Carex* (Cyperaceae, 11 species including seven endemic), *Dendroseris* (Asteraceae, 12 species, all endemic), and *Hymenophyllum* (Hymenophyllaceae, 11 species, only one endemic). Overall, species endemicity is high (65%), placing JF as third among the island systems in terms of species-level endemicity, and equal with New Zealand and the Hawaiian Islands (11%) at the generic-level. Ferns represent about one-quarter of native vascular plant species (57 species, 46% endemic) and genera (24 genera, of which only monotypic *Thyrsopteris* is endemic); there are no native gymnosperms. There are phylogenies for 33 genera but only eight of these are dated. Between 1 and 7 native JF species (20–100%) are included in the phylogenies; 21 genera (63% of those with a phylogeny) have 50% or more species included in phylogenies. Roughly 20% of these genera are monophyletic or nearly so in JF (i.e., one JF origin likely), 20% are not monophyletic in JF (i.e., more than one origin in JF), and the remaining 60% are unknown due to lack of phylogeny or sampling. Only about half of the JF genera have at least one species with a chromosome count, and there are no genome size estimates known. JF genera have either one or two ploidy levels represented on the archipelago, ranging from 2*x* to 8*x*, with diploids and tetraploids the most common. Of the 33 JF genera with phylogenies, about 70% have at least one species with a chromosome count and half have at least 50% species with a chromosome count. Of these, almost all have only one ploidy level.

The Galápagos Islands have the highest number of main islands with an area only slightly greater than the Canary Islands (Table 1). Native vascular genera total 277, seven are endemic (3%); 105 (38%) of these have between 2 and 19 species, and the remaining are monotypic on the archipelago. Ferns represent ca. 23% of vascular plant species (9% of these are endemic); there are no endemic fern genera. Like the Hawaiian and Juan Fernández Islands, there are no native gymnosperms in the Galápagos. Molecular-based phylogenies are available for 42 genera, and only four of these are dated phylogenies. Between 1 and 13 GI species (11–87%) are included in the phylogenies; 21 genera have 50% or more species included in the phylogenies. Chromosome numbers are available for only 21 genera, and of these 13 genera have two or more species. Data for chromosome numbers for native taxa and dated phylogenies were only available for one lineage, which was not included in the statistical analyses. Where chromosome numbers are available, the species are primarily 4*x* or 6*x* with just one ploidy level in those lineages in most cases. In two cases, multiple ploidy levels exist on the archipelago and these were both in fern genera [*Adiantum* (Pteridaceae) and *Polypodium* (Polypodiaceae)].

### Statistical Analysis of Island System Data

Table 2 summarizes the lineages from the four island systems that fitted the criteria for the statistical analyses (i.e., at least two native species on the island system, dated phylogeny available with at least one native island species included, and at least one chromosome count of a native island specimen). New Zealand lineages comprised 66% of the data set, followed by the Hawaiian and Canary Islands with 15% each, and Juan Fernández at 3% (no data were available for the Galápagos Islands). Just under half (45%) of the genera were monophyletic on the island system. For the majority of lineages where the ploidy level of the sister group could be determined, the lowest ploidy level on the island was the same as the sister group (JF 60% *n* = 6, HI 73% *n* = 23, CI 92% *n* = 23, NZ 92% *n* = 98; Table 2). The remaining lineages had a polyploidization event that occurred sometime along the stem lineage immediately before or after colonization of the island and thus may represent island neopolyploidization. New Zealand had the oldest mean stem age (15.63 vs. 4.18million years in Juan Fernández) and the highest number of ploidy levels per lineage (1.7 vs. 1.0 in Juan Fernández). The Hawaiian Islands had the highest mean number of island endemic species per lineage (16.1 vs. 4.8 in Juan Fernández) but also the lowest mean percentage of species with chromosome counts (50 vs. 86% in New Zealand).

Our statistical analysis supported the hypothesis that polyploidy shapes the diversification of island floras (Figure 2). We found that greater levels of polyploidy directly promoted endemic diversity on island systems (P1; *t*_150_ = 3.11, *p* = 0.002). Over the range of observed ploidy levels (1–6), the estimated number of species in island genera increased from a mean of 1 to 4 [95% confidence interval (CI) for increase: 0.7–9.9]. Polyploidy itself was enhanced by a larger source of potential congeneric colonists (P4; *t*_150_ = 5.36, *p* < 0.001) and a change in ploidy level from overseas sister taxa (P3; *t*_150_ = 4.04, *p* < 0.001). Lineages that changed in ploidy near the time of island colonization had, on average, 4.4 ploidy levels as compared with 2.5 levels where these changes were absent (95% CI for difference: 0.83–3.22). In these same lineages, as the size of the potential colonist pool increased from the 25th to 75th percentile of observed values (5–275 species in the source pool), estimated ploidy levels increased from a mean of 3.5 to 5.4 (95% CI for increase: 1.0–2.9).

**Figure 3:**
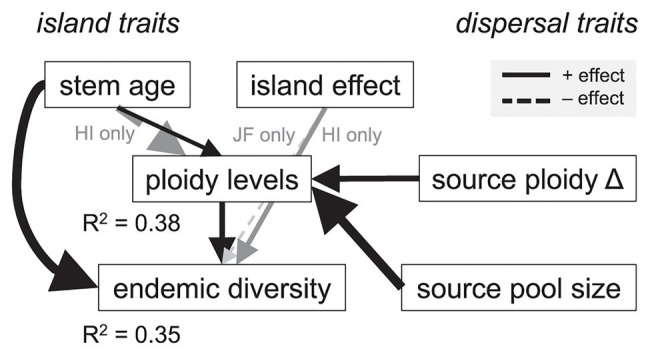
Estimated path analysis to explain the influence of polyploidy on the diversity of endemic island plant species. The model was fitted to 150 lineages across four archipelagos for which we had published estimates of their stem age and chromosome numbers. Arrows are scaled proportional to standardized effect sizes and only statistically significant pathways (*p* < 0.05) are shown (e.g., direct effects of repeat colonization and source pool size on endemic diversity were tested but not significant). Gray text and arrows refer to pathways that were statistically significant only on the Hawaiian (HI) or the Juan Fernández (JF) Islands.

Lineage age also affected diversification outcomes. Older lineages were more diverse (*t*_150_ = 3.69, *p* < 0.001), as expected if they had more time to diversify (P5) and had slightly more ploidy levels across island systems (P2; *t*_150_ = 1.98, *p* = 0.049). Over the interquartile range of observed stem ages, ploidy levels were estimated to increase from a mean of 4.1 to 4.7 (95% CI for increase: 0.4–2.1). The effect of stem age on ploidy levels was, however, reversed (negative and statistically significant) for the Hawaiian Islands (Figure 3, “island effect”), resulting in fewer ploidy levels in the older Hawaiian lineages (*t*_150_ = −2.60, *p* = 0.010). Endemic diversity was also higher, on average, for Hawaiian lineages (*t*_150_ = 2.11, *p* = 0.036) and lower in Juan Fernández lineages (*t*_150_ = −2.10, *p* = 0.038; Figure 3, “island effect”). Whether lineages were monophyletic on the island system (P7) or had more potential congeneric colonists outside of the island system (P6) had no direct effect on endemic diversity (*t*_150_ = 1.62, *p* = 0.107 and *t*_150_ = 1.62, *p* = 0.108, respectively). Tests of directed separation indicated a missing path in our analysis from the lineages being monophyletic (“repeat colonization”) to ploidy levels (“polyploidy,” Figure 2). Including this path in our final model made no difference to our results (*t*_150_ = 0.15, *p* = 0.885). Overall, the model predicted both endemic diversity and the number of ploidy levels reasonably well (*R*^2^ = 0.35 and 0.38, respectively; Figure 3).

## Discussion

Understanding evolutionary processes on islands has improved with molecular phylogenetic analyses and especially the development of time-calibrated phylogenies, which provide a temporal framework for island colonization. Using this information from five island systems, we highlight the important role of polyploidy in the dispersal, colonization, and diversification of island lineages. Our results overturn the perception that chromosomal stasis is a feature of island systems, and instead provide evidence that demonstrates the importance of polyploidy in promoting both colonization and species diversification.

Previous authors have already commented that polyploidy may assist colonization of islands by facilitating the establishment of species (Linder and Barker, 2014) and this also parallels findings that polyploidy is more common in invasive plants (Te Beest et al., 2012). Furthermore, polyploids seem to be preadapted to island colonization. One factor providing polyploids an advantage over related diploids in the colonization of new habitats is higher genetic diversity, which leads to lower inbreeding depression of polyploids (Rosche et al., 2017) and improved adaptability (Scarrow et al., 2020). Other factors include improved environmental tolerance (Moura et al., 2020), higher frequency of vegetative reproduction, and selfing (in part due to a breakdown of self-incompatibility; Robertson et al., 2011), which provide a means to colonize and survive longer at low population size (Baker, 1967; Herben et al., 2017). Thus, there are several arguments for considering polyploids to be superior colonizers but only very few that support the idea that polyploids are better dispersers (e.g., Kuo et al., 2016 for ferns). For example, Harbaugh (2008) suggested that polyploid *Santalum* (Santalaceae) have smaller seeds and fruits with a thicker endocarp, making them easier to disperse by birds. However, easy dispersal does not necessarily translate to frequent establishment and consequently more species on an island.

Our analyses demonstrate that polyploidy contributes significantly to diversification of established island lineages. Endemic richness on islands increases over time in colonizing lineages, reflecting the accumulation of new species through local speciation on islands. The results of the path analysis indicate that polyploidy influences species diversity on islands through colonizing species belonging to large lineages, apparently able to speciate more than others independent of the geographic setting. Thus, the source pool size is one factor that drives polyploidization, which in turn affects endemicity on the archipelago. Additionally, ploidy levels also increase over time and thereby contribute to increasing the number of endemic taxa. According to our path analysis (Figure 3), lineages are more likely to produce new polyploid species on the archipelago, when the lineage already underwent polyploidization after diverging from its mainland sister lineage.

Together, our results agree with previous conclusions that speciation on islands is similar to elsewhere (Takayama et al., 2018) and supports our hypothesis that polyploidy is a diversification trait on islands, with this trait being taxonomically more frequent in some lineages than others (Wood et al., 2009), e.g., Asteraceae (Figure 1). For example, Meudt et al. (2015) demonstrated that diversification in the hexaploid lineage of *Veronica* in New Zealand, the largest endemic lineage in New Zealand (Figure 1), is related to its decrease in genome size. This genome downsizing is also related to diversification in mainland lineages of *Veronica*. A connection of genome downsizing in polyploid lineages has similarly been found in polyploid *Cheirolophus* (Asteraceae) on the Canary Islands (Hidalgo et al., 2017). More generally, Kapralov and Filatov (2011) demonstrated a correlation of low genome size with species richness in endemic island genera. Thus, the relevance of polyploidy for island diversification involves both lineage features, such as the tendency to form polyploids, and aspects of island characteristics, such as habitat heterogeneity and availability of pollinators. Islands also have many intrinsic features that may facilitate polyploid differentiation and persistence, including small size, habitat heterogeneity and proximity, and nutrient-rich soils associated with volcanic activity or maritime animals.

It remains to be studied which aspects of polyploidy are critical for a given taxon and island and whether there is a more general aspect that allows polyploids to disperse, colonize, and/or diversify. Given advances in molecular biology, it seems feasible in the future to determine a propensity to form polyploid species (Bomblies, 2020) and to detect ancient rounds of polyploidy (Tiley et al., 2018). At the moment, polyploidy *per se* outside the island systems could not be considered in our analyses because of the difficulty to establish this in many cases (i.e., where the chromosome number of a sister lineage was unknown) and our observations that many of the lineages colonizing the island systems are already polyploid lineages of mainland taxa [e.g., *Coprosma* (Rubiaceae)].Where there is high species richness within a genus, it seems the polyploid lineage is the one that colonizes the island systems, but overall these examples remain few. One excellent example is the Hawaiian silversword alliance, a recent radiation that includes ~30 species in three endemic genera, which exhibit a variety of growth forms and exploit diverse habitats (Baldwin and Sanderson, 1998). The endemic Hawaiian species are all allotetraploid derivatives from diploid continental ancestors (Carr, 1998; Barrier et al., 1999). Our result of increased diversity on the island systems based on a higher ploidy level of the island lineage compared to the mainland sister lineage, nevertheless, allows us to focus on the question of whether polyploidy is a dispersal or colonization trait. Crawford et al. (2009) already queried whether the colonizers themselves were polyploid or if polyploidy evolved *in situ*, which motivated our first prediction (P1) in our conceptual model. Our literature survey provides many examples of such cases to be studied in the future under different evolutionary scenarios, with and without further diversification, with or without further changes in ploidy level, and in different geological and temporal settings.

### Polyploidy and Diversification on Islands – Generalities

Islands have complex geological histories, and generally New Zealand is considered to be a “continental island” as compared to the other “oceanic islands” that we studied here (Weigelt et al., 2013). However, our analyses showed that this was not a meaningful distinction for determining factors influencing diversification as results from all island systems were generally similar (few island effects, and none that distinguished New Zealand from the other four archipelagos). Thus, despite the fact that New Zealand has not been included with other studies of island polyploid speciation, we find similarities among all the island systems included here in terms of the contribution of polyploidy to species diversification (Tables 1 and 2). Also, the age of New Zealand’s flora, as estimated based on mean stem age per lineage (Table 2), does not distinguish New Zealand from others due to the large variation in age. This large variation in ages of island lineages (Table 2) demonstrates that colonization has been successful mostly independent of time of arrival. Thus, even old islands are dispersal‐ or establishment-limited not niche-limited. Based on Carvajal-Endara et al. (2017) the establishment of a lineage seems to be the bigger hurdle than dispersal. While our study focused on particular island systems that have been well studied, the patterns of species diversification on islands that have emerged may be more general to other island systems (e.g., López-Alvarado et al., 2020). Other island systems would be interesting to evaluate for these larger-scale patterns, as studies on their specific flora are becoming available (e.g., Sardinia – López-Alvarado et al., 2020; Balearic Islands – Rosselló and Castro, 2008). Indeed, Rice et al. (2019), in their global analysis of polyploid biogeography, find high levels of polyploidy on several island systems, which mainly relate to the climatic conditions and predominance of perennial taxa.

### Polyploidy and Species Diversification on the Individual Islands

New Zealand is considered a continental island, because it separated from Gondwana 80 million years ago. Nevertheless, much of its flora, especially non-woody taxa, is considered to have arrived by long distance-dispersal following large-scale marine transgression during the Oligocene (Heenan and McGlone, 2019). Still, lineages on New Zealand included in our analyses are on average older than those of the other island systems (Table 2). Sanmartín et al. (2007) inferred most of the colonization events to have occurred from Australia, and our survey of available phylogenies shows that many New Zealand genera are part of larger Southern Hemisphere lineages. New Zealand has the largest flora of those studied here with a high number of endemics and several large radiations involving polyploid formation (e.g., *Veronica*; Meudt et al., 2015, Table 1). The flora is well-studied phylogenetically and chromosomally with genome size measurements increasing recently, which makes New Zealand an excellent example for studies on the importance of polyploidy.

The Canary Islands are characterized among the five archipelagos as the one closest to a continent, which may explain its low endemicity despite having the highest diversity per square kilometer of all island systems except Juan Fernández (Table 1). Indeed, nearby mainland Europe or Africa seems to be the ancestral areas for most Canary Island genera and species (Sanmartín et al., 2008). This diversity and the ease to access the island system explains why the Canary Islands have become such an important natural laboratory for evolutionary botanists with studies investigating patterns of dispersal and evolution on the archipelago, such as the evolution of woodiness (Böhle et al., 1996; Schüßler et al., 2019), breeding system (Soto-Trejo et al., 2013), and photosynthetic pathways (Mort et al., 2007). Most important in our context is the intensive karyological study of the archipelago (e.g., Suda et al., 2005; Table 1). Interestingly, a number of studies (e.g., Allan et al., 2004) demonstrate that Canary Island taxa occupy similar habitats as compared to their continental relatives, suggesting that their ancestors might have been pre-adapted to occupying particular habitats.

Polyploidy seems to have a larger impact on diversification on New Zealand and the Canary Islands compared to the other island systems, for example in the largest radiation on the Canary Islands, the *Aeonium* alliance (Figure 1), or *Sideritis* (Lamiaceae; Raskina et al., 2008). The suggestion that polyploidy seems to be less common in Asteraceae on the Canary Islands compared to other island systems (Crawford et al., 2009) only holds for the two largest genera of Asteraceae on the island system (*Argyranthemum*, *Sonchus* alliance), not generally across the family. More detailed analysis on the distribution of polyploids on New Zealand and the Canary Islands will be necessary to determine whether, for example, the larger altitudinal range on these two archipelagos promote diversification by polyploidy. This should be analyzed in connection with the report of the low genome size of the Macaronesian flora in relation with the rest of the world (Suda et al., 2005).

The Hawaiian Islands are the island system farthest away from a continent, and thus its genera are mostly considered to have colonized the island system only once (Funk and Wagner, 1995; Keeley and Funk, 2011). Following from this it also has the highest number of endemics (Table 1). Ancestors of Hawaiian taxa are derived from diverse geographical regions including North America, Australia, Asia, Africa, and other island systems in the Pacific (e.g., New Zealand; Keeley and Funk, 2011; Price and Wagner, 2018). The considerable age of some of its lineages is likely caused by the influence of early colonization of now succumbed islands of the archipelago (García-Verdugo et al., 2019), while most others represent more recent arrivals (Price and Clague, 2002). Despite great interest in the flora of the island system, it is only poorly investigated karyologically (Table 1). Thus, the hypothesis of chromosomal stasis of lineages on the Hawaiian Islands (Stuessy and Crawford, 1998) is founded on few, though important, cases. Nevertheless, the data available suggest a lower importance of polyploidy than in New Zealand and the Canary Islands, chromosomal stasis thus being peculiar to the Hawaiian Islands. One notable pattern for the Hawaiian Islands is the formation of dysploid series (Carr, 1998), which has also been noted on rare occasions in the Canary Islands (*Sideritis*, Barber et al., 2000) and New Zealand (*Veronica*, Wagstaff and Garnock-Jones, 1998).

The Juan Fernández Islands are the smallest archipelago but the flora has been intensively investigated floristically (Greimler et al., 2013) and evolutionarily (Takayama et al., 2018), including a number of karyological studies but so far no measurements of genome sizes (Table 1). The closest continental source is South America, which seems to be the ancestral area for several Juan Fernández genera and species (Stuessy et al., 2017). Despite the fact that some genera have species with different ploidy levels on different islands, there is no clear case of polyploid origin there because those cases are either based on separate colonization events or have not been studied phylogenetically. Also, Stuessy et al. (2017) state only two possible cases of polyploid origins on this island system. The high number of polyploids in the ancestral lineages of some Juan Fernández endemics, such as *Dendroseris* and *Erigeron* (Asteraceae), therefore suggests that polyploidy is a dispersal or colonization trait rather than a diversification trait for the flora of Juan Fernández. Two factors could explain this lack of polyploid speciation on Juan Fernández Islands: their small size providing little space for the avoidance of inter-cytotype gene flow or the young age of the lineages compared to that of the other islands (Table 2). Given more time the intraspecific variation in ploidy of several species on the islands [Stuessy et al., 2017; e.g., in *Eryngium bupleuroides* (Asteraceae); Figure 1] could translate into new polyploid species.

The Galápagos have not been included in our analyses, despite the importance of this island system for the history of evolution and biogeography, because its flora has been poorly studied. Its flora, like its fauna, is considered to be derived from South America (Darwin, 1839; Elisens, 1992), although exceptions occur (e.g., Andrus et al., 2009). The Galápagos flora is only a third the size of the Canary Islands flora despite similar area, possibly due to its dryness, which could also explain the low number of endemic fern species (Table 1). Polyploid speciation, however, has been shown to occur in the genus *Pectis* (Asteraceae, Hansen et al., 2016). In *Scalesia* (Asteraceae), the largest radiation on the archipelago, the species are considered to be tetraploid but have not further diversified in ploidy level (Eliasson, 1974; Fernández-Mazuecos et al., 2020).

The discussion of these five island systems already demonstrates that there are as many idiosyncratic patterns as there are island systems, but polyploidy seems to play a role in many of them. For example, Rosselló and Castro (2008) found a large frequency of neopolyploidization events among the endemic plants of the Balearic Islands, whereas Sun and Stuessy (1998) recorded chromosomal stasis in the flora of Ullung Islands. Studies of additional island systems that vary in their locations and floristic complexities would be beneficial for enhancing our understanding of endemic polyploid diversification.

## Outlook and Future Directions

We see the study of polyploidy on islands as an exciting avenue to further our understanding of polyploid species diversification. Our comparative study was limited to data that were available for the island systems under study and we were surprised that some of these “classic” island systems remain poorly known chromosomally and phylogenetically, especially the Galápagos. While genomic studies have revolutionized our ideas of polyploid genome dynamics (e.g., Bomblies, 2020), there is much value in continuing to generate chromosome numbers and dated phylogenies for native island endemics. Because island radiations are often young and/or rapid on an evolutionary timescale, in many cases acquiring a well-resolved phylogeny for a group of species based on single or few gene sequences has been challenging (e.g., Meudt and Simpson, 2006; Knope et al., 2012; Vitales et al., 2014). Newer methodologies that take advantage of next-generation sequencing methods should be helpful in this regard (Larridon et al., 2020). Similarly, overseas sister lineages need to be investigated to understand the context of island diversification. One additional limitation to our study was the lack of data for genome size estimates, either to help resolve ploidy levels or to analyze its effect on polyploid species diversification on the island systems. Given that genome downsizing is considered to be an important part of polyploid evolution (Soltis et al., 2016), we see this factor as a potentially important player in facilitating establishment on islands, as mentioned previously for *Veronica* (Meudt et al., 2015). Additionally, future studies should further compare the effect of polyploidy on colonization and diversification among woody vs. herbaceous, dry vs. fleshy fruited, and selfing vs. outcrossing lineages (Vamosi et al., 2007) for a more complete picture of island diversification.

## Data Availability Statement

The original contributions presented in the study are included in the article/Supplementary Material, further inquiries can be directed to the corresponding authors.

## Author Contributions

HMM, JAT, AJT, DCA, and WGL designed the study and compiled the data, with assistance from SCN, AJB and JI. AJT and JI completed statistical analyses. All authors contributed to drafting the manuscript and developing the final version and are accountable for the contents of the work.

### Conflict of Interest

The authors declare that the research was conducted in the absence of any commercial or financial relationships that could be construed as a potential conflict of interest.

## Footnotes

^1^http://legacy.tropicos.org/Project/IPCN

^2^http://ccdb.tau.ac.il/

^3^https://cvalues.science.kew.org/

^4^https://automeris.io/WebPlotDigitizer/

## Supplementary Material

Supplementary File 1 The data file analyzed in this study, which includes the following information from 150 island lineages from New Zealand (n = 98), Canary Islands (n = 23), Hawaiian Islands (n = 23), and Juan Fernández Islands (n = 6): lineage (genus) name, number of endemic species, stem age, number of ploidy levels, whether ploidy level differs from closest non-island sister group, monophyly on the island, and number of species in closest non-island sister group.

Supplementary File 2 References for the 150 lineages included in the statistical analyses (with dated phylogenies) grouped by island system.
