# Supplementary material for: Polyploidy on Islands: Its Emergence and Importance for Diversification
Source: Front Plant Sci. 2021 Mar 4;12:637214. doi: 10.3389/fpls.2021.637214 (PMC7982887; doi:10.3389/fpls.2021.637214)
Supplement: Supplementary File 2 — References for the 150 lineages included in the statistical analyses (with dated phylogenies) grouped by island system. [file Data_Sheet_2.docx]

Meudt et al. 2021. Polyploidy on islands: its emergence and importance for diversification. Frontiers in Plant Science. DOI: 10.3389/fpls.2021.637214

**Supplementary File 2**. References for the 150 lineages included in the statistical analyses (with dated phylogenies) grouped by island system.

**Canary Islands**

Albaladejo, R.G., Martín-Hernanz, S., Reyes-Betancort, J.A., Santos-Guerra, A., Olangua-Corral, M., and Aparicio, A. (2020). Reconstruction of the spatio-temporal diversification and ecological niche evolution of *Helianthemum* (Cistaceae) in the Canary Islands using Genotyping-by-Sequencing data. *Annals of Botany* in press. doi: 10.1093/aob/mcaa090. .

Banasiak, Ł., Piwczyński, M., Uliński, T., Downie, S.R., Watson, M.F., Shakya, B., et al. (2013). Dispersal patterns in space and time: a case study of Apiaceae subfamily Apioideae. *Journal of Biogeography* 40(7)**,** 1324-1335.

Calleja, J.A., Garcia-Jacas, N., Roquet, C., and Susanna, A. (2016). Beyond the Rand Flora pattern: Phylogeny and biogeographical history of *Volutaria* (Compositae). *Taxon* 65(2)**,** 315-332.

Caujapé-Castells, J., Jansen, R.K., Membrives, N., Pedrola-Monfort, J., Montserrat, J.M., and Ardanuy, A. (2001). Historical biogeography of *Androcymbium* willd. (Colchicaceae) in Africa: evidence from cpDNA RFLPs. *Botanical Journal of the Linnean Society* 136(4)**,** 379-392.

Cerros‐Tlatilpa, R., Columbus, J.T., and Barker, N.P. (2011). Phylogenetic relationships of *Aristida* and relatives (Poaceae, Aristidoideae) based on noncoding chloroplast (*trnL‐F*, *rpl16*) and nuclear (ITS) DNA sequences. *American Journal of Botany* 98(11)**,** 1868-1886.

Couvreur, T.L., Franzke, A., Al-Shehbaz, I.A., Bakker, F.T., Koch, M.A., and Mummenhoff, K. (2010). Molecular phylogenetics, temporal diversification, and principles of evolution in the mustard family (Brassicaceae). *Molecular Biology and Evolution* 27(1)**,** 55-71.

Désamoré, A., Laenen, B., Devos, N., Popp, M., González‐Mancebo, J.M., Carine, M.A., et al. (2011). Out of Africa: north‐westwards Pleistocene expansions of the heather *Erica arborea*. *Journal of Biogeography* 38(1)**,** 164-176.

Dias, E.F., Kilian, N., Silva, L., Schaefer, H., Carine, M., Rudall, P.J., et al. (2018). Phylogeography of the Macaronesian lettuce species *Lactuca watsoniana* and *L. palmensis* (Asteraceae). *Biochemical Genetics* 56(4)**,** 315-340.

Francisco‐Ortega, J., Santos‐Guerra, A., Hines, A., and Jansen, R.K. (1997). Molecular evidence for a Mediterranean origin of the Macaronesian endemic genus *Argyranthemum* (Asteraceae). *American Journal of Botany* 84(11)**,** 1595-1613.

Friesen, N., Herden, T., and Schoenfelder, P. (2015). *Allium canariense* (Amaryllidaceae), a species endemic to the Canary Islands. *Phytotaxa* 221(1)**,** 1-20.

García-Maroto, F., Mañas-Fernández, A., Garrido-Cárdenas, J.A., Alonso, D.L., Guil-Guerrero, J.L., Guzmán, B., et al. (2009). Δ6-Desaturase sequence evidence for explosive Pliocene radiations within the adaptive radiation of Macaronesian *Echium* (Boraginaceae). *Molecular Phylogenetics and Evolution* 52(3)**,** 563-574.

Guzmán, B., and Vargas, P. (2010). Unexpected synchronous differentiation in Mediterranean and Canarian *Cistus* (Cistaceae). *Perspectives in Plant Ecology, Evolution and Systematics* 12(3)**,** 163-174.

Inda, L.A., Sanmartín, I., Buerki, S., and Catalán, P. (2014). Mediterranean origin and Miocene–Holocene Old World diversification of meadow fescues and ryegrasses (*Festuca* subgenus *Schedonorus* and *Lolium*). *Journal of Biogeography* 41(3)**,** 600-614.

Inda, L.A., Segarra-Moragues, J.G., Müller, J., Peterson, P.M., and Catalán, P. (2008). Dated historical biogeography of the temperate Loliinae (Poaceae, Pooideae) grasses in the northern and southern hemispheres. *Molecular Phylogenetics and Evolution* 46(3)**,** 932-957.

Jones, K.E., Reyes‐Betancort, J.A., Hiscock, S.J., and Carine, M.A. (2014). Allopatric diversification, multiple habitat shifts, and hybridization in the evolution of *Pericallis* (Asteraceae), a Macaronesian endemic genus. *American Journal of Botany* 101(4)**,** 637-651.

Kadereit, G., Mavrodiev, E.V., Zacharias, E.H., and Sukhorukov, A.P. (2010). Molecular phylogeny of Atripliceae (Chenopodioideae, Chenopodiaceae): implications for systematics, biogeography, flower and fruit evolution, and the origin of C4 photosynthesis. *American Journal of Botany* 97(10)**,** 1664-1687.

Kim, S.-C., McGowen, M.R., Lubinsky, P., Barber, J.C., Mort, M.E., and Santos-Guerra, A. (2008). Timing and tempo of early and successive adaptive radiations in Macaronesia. *PLoS One* 3(5)**,** e2139.

Kondraskov, P., Schütz, N., Schüßler, C., de Sequeira, M.M., Guerra, A.S., Caujapé-Castells, J., et al. (2015). Biogeography of Mediterranean hotspot biodiversity: re-evaluating the 'Tertiary relict' hypothesis of Macaronesian laurel forests. *PLoS One* 10(7)**,** e0132091.

Lledó, M.D., Crespo, M.B., Fay, M.F., and Chase, M.W. (2005). Molecular phylogenetics of *Limonium* and related genera (Plumbaginaceae): biogeographical and systematic implications. *American Journal of Botany* 92(7)**,** 1189-1198.

Martín‐Bravo, S., Jiménez‐Mejías, P., Villaverde, T., Escudero, M., Hahn, M., Spalink, D., et al. (2019). A tale of worldwide success: Behind the scenes of *Carex* (Cyperaceae) biogeography and diversification. *Journal of Systematics and Evolution* 57(6)**,** 695-718.

Meseguer, A.S., Aldasoro, J.J., and Sanmartín, I. (2013). Bayesian inference of phylogeny, morphology and range evolution reveals a complex evolutionary history in St. John’s wort (*Hypericum*). *Molecular Phylogenetics and Evolution* 67(2)**,** 379-403.

Míguez, M., Gehrke, B., Maguilla, E., Jiménez-Mejías, P., and Martín-Bravo, S. (2017). *Carex* sect. *Rhynchocystis* (Cyperaceae): a Miocene subtropical relict in the Western Palaearctic showing a dispersal-derived Rand Flora pattern. *Journal of Biogeography* 44(10)**,** 2211-2224. doi: <https://doi.org/10.1111/jbi.13027>.

Olesen, J.M., Alarcón, M., Ehlers, B.K., Aldasoro, J.J., and Roquet, C. (2012). Pollination, biogeography and phylogeny of oceanic island bellflowers (Campanulaceae). *Perspectives in Plant Ecology, Evolution and Systematics* 14(3)**,** 169-182.

Papadopulos, A.S., Baker, W.J., Crayn, D., Butlin, R.K., Kynast, R.G., Hutton, I., et al. (2011). Speciation with gene flow on Lord Howe Island. *Proceedings of the National Academy of Sciences U.S.A.* 108(32)**,** 13188-13193.

Pimentel, M., Sahuquillo, E., Torrecilla, Z., Popp, M., Catalán, P., and Brochmann, C. (2013). Hybridization and long-distance colonization at different time scales: towards resolution of long-term controversies in the sweet vernal grasses (*Anthoxanthum*). *Annals of Botany* 112(6)**,** 1015-1030.

Puppo, P., Curto, M., Velo‐Antón, G., Pérez de Paz, P.L., and Meimberg, H. (2014). The influence of geological history on diversification in insular species: genetic and morphological patterns of *Micromeria* Benth. (Lamiaceae) in Tenerife (Canary archipelago). *Journal of Biogeography* 41(10)**,** 1871-1882.

Romeiras, M.M., Vieira, A., Silva, D.N., Moura, M., Santos-Guerra, A., Batista, D., et al. (2016). Evolutionary and biogeographic insights on the Macaronesian *Beta-Patellifolia* species (Amaranthaceae) from a time-scaled molecular phylogeny. *PLoS One* 11(3)**,** e0152456.

Salvo, G., Ho, S.Y., Rosenbaum, G., Ree, R., and Conti, E. (2010). Tracing the temporal and spatial origins of island endemics in the Mediterranean region: a case study from the citrus family (*Ruta* L., Rutaceae). *Systematic Biology* 59(6)**,** 705-722.

Sessa, E.B., Juslén, A., Väre, H., and Chambers, S.M. (2017). Into Africa: Molecular phylogenetics and historical biogeography of sub‐Saharan African woodferns (*Dryopteris*). *American Journal of Botany* 104(3)**,** 477-486.

Smissen, R., Garnock-Jones, P., and Chambers, G. (2003). Phylogenetic analysis of ITS sequences suggests a Pliocene origin for the bipolar distribution of *Scleranthus* (Caryophyllaceae). *Australian Systematic Botany* 16(3)**,** 301-315.

Valtuena, F.J., Rodríguez-Riaño, T., Lopez, J., Mayo, C., and Ortega-Olivencia, A. (2017). Peripatric speciation in an endemic Macaronesian plant after recent divergence from a widespread relative. *PloS One* 12(6)**,** e0178459.

Villaverde, T., Pokorny, L., Olsson, S., Rincón‐Barrado, M., Johnson, M.G., Gardner, E.M., et al. (2018). Bridging the micro‐and macroevolutionary levels in phylogenomics: Hyb‐Seq solves relationships from populations to species and above. *New Phytologist* 220(2)**,** 636-650.

Vitales, D., Garnatje, T., Pellicer, J., Vallès, J., Santos-Guerra, A., and Sanmartín, I. (2014). The explosive radiation of *Cheirolophus* (Asteraceae, Cardueae) in Macaronesia. *BMC Evolutionary Biology* 14(1)**,** 1-15.

**Hawaiian Islands**

Appelhans, M.S., Reichelt, N., Groppo, M., Paetzold, C., and Wen, J. (2018). Phylogeny and biogeography of the pantropical genus *Zanthoxylum* and its closest relatives in the proto-Rutaceae group (Rutaceae). *Molecular Phylogenetics and Evolution* 126**,** 31-44.

Areces-Berazain, F., and Ackerman, J. (2016). Phylogenetics, delimitation and historical biogeography of the pantropical tree genus *Thespesia* (Malvaceae, Gossypieae). *Botanical Journal of the Linnean Society* 181(2)**,** 171-198.

Bacon, C.D., Baker, W.J., and Simmons, M.P. (2012). Miocene dispersal drives island radiations in the palm tribe Trachycarpeae (Arecaceae). *Systematic Biology* 61(3)**,** 426-442.

Besnard, G., Rubio de Casas, R., Christin, P.-A., and Vargas, P. (2009). Phylogenetics of *Olea* (Oleaceae) based on plastid and nuclear ribosomal DNA sequences: tertiary climatic shifts and lineage differentiation times. *Annals of Botany* 104(1)**,** 143-160.

Birch, J.L., Keeley, S.C., and Morden, C.W. (2012). Molecular phylogeny and dating of Asteliaceae (Asparagales): *Astelia* s.l. evolution provides insight into the Oligocene history of New Zealand. *Molecular Phylogenetics and Evolution* 65(1)**,** 102-115.

Cantley, J.T., Markey, A.S., Swenson, N.G., and Keeley, S.C. (2016). Biogeography and evolutionary diversification in one of the most widely distributed and species rich genera of the Pacific. *AoB Plants* 8.

Chacón, J., Madriñán, S., Chase, M.W., and Bruhl, J.J. (2006). Molecular phylogenetics of *Oreobolus* (Cyperaceae) and the origin and diversification of the American species. *Taxon* 55(2)**,** 359-366.

Chao, Y.-S., Rouhan, G., Amoroso, V.B., and Chiou, W.-L. (2014). Molecular phylogeny and biogeography of the fern genus *Pteris* (Pteridaceae). *Annals of Botany* 114(1)**,** 109-124.

Ganders, F.R., Berbee, M., and Pirseyedi, M. (2000). ITS base sequence phylogeny in *Bidens* (Asteraceae): evidence for the continental relatives of Hawaiian and Marquesan *Bidens*. *Systematic Botany* 25(1)**,** 122-133.

Givnish, T.J., Bean, G.J., Ames, M., Lyon, S.P., and Sytsma, K.J. (2013). Phylogeny, floral evolution, and inter-island dispersal in Hawaiian *Clermontia* (Campanulaceae) based on ISSR variation and plastid spacer sequences. *PLoS One* 8(5)**,** e62566.

Givnish, T.J., Millam, K.C., Mast, A.R., Paterson, T.B., Theim, T.J., Hipp, A.L., et al. (2009). Origin, adaptive radiation and diversification of the Hawaiian lobeliads (Asterales: Campanulaceae). *Proceedings of the Royal Society B: Biological Sciences* 276(1656)**,** 407-416.

Harrington, M.G., and Gadek, P.A. (2009). A species well travelled–the *Dodonaea viscosa* (Sapindaceae) complex based on phylogenetic analyses of nuclear ribosomal ITS and ETSf sequences. *Journal of Biogeography* 36(12)**,** 2313-2323.

Hauenschild, F., Favre, A., Schulz, M., and Muellner-Riehl, A.N. (2018). Biogeographic analyses support an Australian origin for the Indomalesian-Australasian wet forest-adapted tropical tree and shrub genus *Alphitonia* and its close allies (Rhamnaceae). *Botanical Journal of the Linnean Society* 188(1)**,** 1-20.

Hummer, K.E., Carter, K.A., Liston, A., Bassil, N.V., Alice, L.A., Bushakra, J.M., et al. (2019). Target capture sequencing unravels *Rubus* evolution. *Frontiers in Plant Science* 10**,** 1615.

Johnson, M.A., Clark, J.R., Wagner, W.L., and McDade, L.A. (2017). A molecular phylogeny of the Pacific clade of *Cyrtandra* (Gesneriaceae) reveals a Fijian origin, recent diversification, and the importance of founder events. *Molecular Phylogenetics and Evolution* 116**,** 30-48.

Knope, M.L., Morden, C.W., Funk, V.A., and Fukami, T. (2012). Area and the rapid radiation of Hawaiian *Bidens* (Asteraceae). *Journal of Biogeography* 39(7)**,** 1206-1216.

Kuo, L.-Y., Ebihara, A., Shinohara, W., Rouhan, G., Wood, K.R., Wang, C.-N., et al. (2016). Historical biogeography of the fern genus *Deparia* (Athyriaceae) and its relation with polyploidy. *Molecular Phylogenetics and Evolution* 104**,** 123-134.

Landis, M.J., Freyman, W.A., and Baldwin, B.G. (2018). Retracing the Hawaiian silversword radiation despite phylogenetic, biogeographic, and paleogeographic uncertainty. *Evolution* 72(11)**,** 2343-2359.

Lim, J.Y., Marshall, C.R., Zimmer, E.A., and Wagner, W.L. (2019). Multiple colonizations of the Pacific by *Peperomia* (Piperaceae): Complex patterns of long‐distance dispersal and parallel radiations on the Hawaiian Islands. *Journal of Biogeography* 46(12)**,** 2651-2662.

Marcussen, T., Jakobsen, K.S., Danihelka, J., Ballard, H.E., Blaxland, K., Brysting, A.K., et al. (2012). Inferring species networks from gene trees in high-polyploid North American and Hawaiian violets (*Viola*, Violaceae). *Systematic Biology* 61(1)**,** 107-126.

Marcussen, T., and Meseguer, A.S. (2017). Species-level phylogeny, fruit evolution and diversification history of *Geranium* (Geraniaceae). *Molecular Phylogenetics and Evolution* 110**,** 134-149.

Morden, C.W., Gardner, D.E., and Weniger, D.A. (2003). Phylogeny and biogeography of Pacific *Rubus* subgenus *Idaeobatus* (Rosaceae) species: Investigating the origin of the endemic Hawaiian raspberry *R. macraei*. *Pacific Science* 57(2)**,** 181-197.

Ocampo, G., and Columbus, J.T. (2012). Molecular phylogenetics, historical biogeography, and chromosome number evolution of *Portulaca* (Portulacaceae). *Molecular Phylogenetics and Evolution* 63(1)**,** 97-112.

Pryer, K.M., Schuettpelz, E., Wolf, P.G., Schneider, H., Smith, A.R., and Cranfill, R. (2004). Phylogeny and evolution of ferns (monilophytes) with a focus on the early leptosporangiate divergences. *American Journal of Botany* 91(10)**,** 1582-1598.

Razafimandimbison, S.G., Kainulainen, K., Wikström, N., and Bremer, B. (2017). Historical biogeography and phylogeny of the pantropical Psychotrieae alliance (Rubiaceae), with particular emphasis on the Western Indian Ocean Region. *American Journal of Botany* 104(9)**,** 1407-1423.

Sebastian, P., Schaefer, H., Lira, R., Telford, I.R., and Renner, S.S. (2012). Radiation following long‐distance dispersal: the contributions of time, opportunity and diaspore morphology in *Sicyos* (Cucurbitaceae). *Journal of Biogeography* 39(8)**,** 1427-1438.

Snak, C., Vatanparast, M., Silva, C., Lewis, G.P., Lavin, M., Kajita, T., et al. (2016). A dated phylogeny of the papilionoid legume genus *Canavalia* reveals recent diversification by a pantropical liana lineage. *Molecular Phylogenetics and Evolution* 98**,** 133-146.

Sundue, M.A., Parris, B.S., Ranker, T.A., Smith, A.R., Fujimoto, E.L., Zamora-Crosby, D., et al. (2014). Global phylogeny and biogeography of grammitid ferns (Polypodiaceae). *Molecular Phylogenetics and Evolution* 81**,** 195-206.

Willyard, A., Wallace, L.E., Wagner, W.L., Weller, S.G., Sakai, A.K., and Nepokroeff, M. (2011). Estimating the species tree for Hawaiian *Schiedea* (Caryophyllaceae) from multiple loci in the presence of reticulate evolution. *Molecular Phylogenetics and Evolution* 60(1)**,** 29-48.

Xu, K.W., Zhang, L., Rothfels, C.J., Smith, A.R., Viane, R., Lorence, D., et al. (2020). A global plastid phylogeny of the fern genus *Asplenium* (Aspleniaceae). *Cladistics* 36(1)**,** 22-71.

**Juan Fernández**

Bräuchler, C., Meimberg, H., and Heubl, G. (2010). Molecular phylogeny of Menthinae (Lamiaceae, Nepetoideae, Mentheae)–taxonomy, biogeography and conflicts. *Molecular Phylogenetics and Evolution* 55(2)**,** 501-523.

Crawford, D.J., Stuessy, T.F., Cosner, M.B., Haines, D.W., Silva, M., and Baeza, M. (1992). Evolution of the genus *Dendroseris* (Asteraceae: Lactuceae) on the Juan Fernández Islands: evidence from chloroplast and ribosomal DNA. *Systematic Botany***,** 676-682.

Drew, B.T., and Sytsma, K.J. (2012). Phylogenetics, biogeography, and staminal evolution in the tribe Mentheae (Lamiaceae). *American Journal of Botany* 99(5)**,** 933-953.

Iwanycki Ahlstrand, N., Verstraete, B., Hassemer, G., Dunbar‐Co, S., Hoggard, R., Meudt, H., et al. (2019). Ancestral range reconstruction of remote oceanic island species of *Plantago* (Plantaginaceae) reveals differing scales and modes of dispersal. *Journal of Biogeography* 46(4)**,** 706-722.

Lim, J.Y., Marshall, C.R., Zimmer, E.A., and Wagner, W.L. (2019). Multiple colonizations of the Pacific by *Peperomia* (Piperaceae): Complex patterns of long‐distance dispersal and parallel radiations on the Hawaiian Islands. *Journal of Biogeography* 46(12)**,** 2651-2662.

Martín-Bravo, S., Jiménez-Mejías, P., Villaverde, T., Escudero, M., Hahn, M., Spalink, D., et al. (2019). A tale of worldwide success: Behind the scenes of *Carex* (Cyperaceae) biogeography and diversification. *Journal of Systematics and Evolution* 57(6)**,** 695-718. doi: <https://doi.org/10.1111/jse.12549>.

Ruiz, E., Crawford, D.J., Stuessy, T.F., González, F., Samuel, R., Becerra, J., et al. (2004). Phylogenetic relationships and genetic divergence among endemic species of *Berberis, Gunnera, Myrceugenia* and *Sophora* of the Juan Fernández Islands (Chile) and their continental progenitors based on isozymes and nrITS sequences. *Taxon* 53(2)**,** 321-332.

Ruiz, E., Marticorena, C., Crawford, D., Stuessy, T., González, F., Montoya, R., et al. (2000). Morphological and ITS sequence divergence between taxa of *Cuminia* (Lamiaceae), an endemic genus of the Juan Fernández Islands, Chile. *Brittonia* 52(4)**,** 341-350.

Trusty, J.L., Olmstead, R.G., Bogler, D.J., Santos-Guerra, A., and Francisco-Ortega, J. (2004). Using molecular data to test a biogeographic connection of the Macaronesian genus *Bystropogon* (Lamiaceae) to the New World: a case of conflicting phylogenies. *Systematic Botany* 29(3)**,** 702-715.

Wanntorp, L., Wanntorp, H.-E., and Källersjö, M. (2002). Phylogenetic relationships of *Gunnera* based on nuclear ribosomal DNA ITS region, *rbcL* and *rps16* intron sequences. *Systematic Botany* 27(3)**,** 512-521.

**New Zealand**

Antonelli, A., Humphreys, A.M., Lee, W.G., and Linder, H.P. (2011). Absence of mammals and the evolution of New Zealand grasses. *Proceedings of the Royal Society B: Biological Sciences* 278(1706)**,** 695-701. doi: doi:10.1098/rspb.2010.1145.

Atherton, R., Lockhart, P., McLenachan, P., de Lange, P., Wagstaff, S., and Shepherd, L. (2015). A molecular investigation into the origin and relationships of karaka/kōpi (*Corynocarpus laevigatus*) in New Zealand. *Journal of the Royal Society of New Zealand* 45(4)**,** 212-220.

Banasiak, Ł., Piwczyński, M., Uliński, T., Downie, S.R., Watson, M.F., Shakya, B., et al. (2013). Dispersal patterns in space and time: a case study of Apiaceae subfamily Apioideae. *Journal of Biogeography* 40(7)**,** 1324-1335.

Berry, P.E., Hahn, W.J., Sytsma, K.J., Hall, J.C., and Mast, A. (2004). Phylogenetic relationships and biogeography of *Fuchsia* (Onagraceae) based on noncoding nuclear and chloroplast DNA data. *American Journal of Botany* 91(4)**,** 601-614.

Biffin, E., Conran, J.G., and Lowe, A.J. (2011). Podocarp evolution: a molecular phylogenetic perspective. *Smithsonian Contributions to Botany*. 1–20. doi: <https://doi.org/10.5479/si.0081024X.95.1>

Biffin, E., Hill, R.S., and Lowe, A.J. (2010a). Did kauri (*Agathis*: Araucariaceae) really survive the Oligocene drowning of New Zealand? *Systematic Biology* 59(5)**,** 594-602.

Biffin, E., Lucas, E.J., Craven, L.A., Ribeiro da Costa, I., Harrington, M.G., and Crisp, M.D. (2010b). Evolution of exceptional species richness among lineages of fleshy-fruited Myrtaceae. *Annals of Botany* 106(1)**,** 79-93.

Birch, J.L., Cantrill, D.J., Walsh, N.G., and Murphy, D.J. (2014). Phylogenetic investigation and divergence dating of *Poa* (Poaceae, tribe Poeae) in the Australasian region. *Botanical Journal of the Linnean Society* 175(4)**,** 523-552.

Birch, J.L., and Keeley, S.C. (2013). Dispersal pathways across the Pacific: the historical biogeography of *Astelia* s.l. (Asteliaceae, Asparagales). *Journal of Biogeography* 40(10)**,** 1914-1927.

Bleeker, W., Weber‐Sparenberg, C., and Hurka, H. (2002). Chloroplast DNA variation and biogeography in the genus *Rorippa* Scop.(Brassicaceae). *Plant Biology* 4(1)**,** 104-111.

Cantley, J.T., Markey, A.S., Swenson, N.G., and Keeley, S.C. (2016). Biogeography and evolutionary diversification in one of the most widely distributed and species rich genera of the Pacific. *AoB Plants* 8.

Chacón, J., Madriñán, S., Chase, M.W., and Bruhl, J.J. (2006). Molecular phylogenetics of *Oreobolus* (Cyperaceae) and the origin and diversification of the American species. *Taxon* 55(2)**,** 359-366.

Cook, L.G., and Crisp, M.D. (2005). Not so ancient: the extant crown group of *Nothofagus* represents a post-Gondwanan radiation. *Proceedings of the Royal Society B: Biological Sciences* 272(1580)**,** 2535-2544.

Crayn, D.M., Rossetto, M., and Maynard, D.J. (2006). Molecular phylogeny and dating reveals an Oligo‐Miocene radiation of dry‐adapted shrubs (former Tremandraceae) from rainforest tree progenitors (Elaeocarpaceae) in Australia. *American Journal of Botany* 93(9)**,** 1328-1342.

Davis, C.C., Webb, C.O., Wurdack, K.J., Jaramillo, C.A., and Donoghue, M.J. (2005). Explosive radiation of Malpighiales supports a mid-Cretaceous origin of modern tropical rain forests. *The American Naturalist* 165(3)**,** E36-E65.

Ford, K.A., Ward, J.M., Smissen, R.D., Wagstaff, S.J., and Breitwieser, I. (2007). Phylogeny and biogeography of *Craspedia* (Asteraceae: Gnaphalieae) based on ITS, ETS and *psbA-trnH* sequence data. *Taxon* 56(3)**,** 783-794. doi: 10.2307/25065861.

Foster, C.S., Ho, S.Y., Conn, B.J., and Henwood, M.J. (2014). Molecular systematics and biogeography of *Logania* R. Br.(Loganiaceae). *Molecular phylogenetics and evolution* 78**,** 324-333.

Givnish, T.J., Pires, J.C., Graham, S.W., McPherson, M.A., Prince, L.M., Patterson, T.B., et al. (2006). Phylogenetic relationships of monocots based on the highly informative plastid gene *ndhF*. *Aliso: A Journal of Systematic and Evolutionary Botany* 22(1)**,** 28-51.

Goldblatt, P., Rodriguez, A., Powell, M., Davies, J.T., Manning, J.C., Van der Bank, M., et al. (2008). Iridaceae 'out of Australasia'? Phylogeny, biogeography, and divergence time based on plastid DNA sequences. *Systematic Botany* 33(3)**,** 495-508.

Gussarova, G., Popp, M., Vitek, E., and Brochmann, C. (2008). Molecular phylogeny and biogeography of the bipolar *Euphrasia* (Orobanchaceae): recent radiations in an old genus. *Molecular Phylogenetics and Evolution* 48(2)**,** 444-460.

Himmelreich, S., Breitwieser, I., and Oberprieler, C. (2012). Phylogeny, biogeography, and evolution of sex expression in the southern hemisphere genus *Leptinella* (Compositae, Anthemideae). *Molecular Phylogenetics and Evolution* 65(2)**,** 464-481.

Hurr, K.A., Lockhart, P.J., Heenan, P.B., and Penny, D. (1999). Evidence for the recent dispersal of *Sophora* (Leguminosae) around the Southern Oceans: molecular data. *Journal of Biogeography* 26(3)**,** 565-577. doi: <https://doi.org/10.1046/j.1365-2699.1999.00302.x>.

Inda, L.A., Segarra-Moragues, J.G., Müller, J., Peterson, P.M., and Catalán, P. (2008). Dated historical biogeography of the temperate Loliinae (Poaceae, Pooideae) grasses in the northern and southern hemispheres. *Molecular Phylogenetics and Evolution* 46(3)**,** 932-957.

Joly, S., Heenan, P.B., and Lockhart, P.J. (2009). A Pleistocene inter-tribal allopolyploidization event precedes the species radiation of *Pachycladon* (Brassicaceae) in New Zealand. *Molecular Phylogenetics and Evolution* 51(2)**,** 365-372.

Knapp, M., Stöckler, K., Havell, D., Delsuc, F., Sebastiani, F., and Lockhart, P.J. (2005). Relaxed molecular clock provides evidence for long-distance dispersal of *Nothofagus* (southern beech). *PLoS Biol* 3(1)**,** e14.

Knopf, P., Schulz, C., Little, D.P., Stützel, T., and Stevenson, D.W. (2012). Relationships within Podocarpaceae based on DNA sequence, anatomical, morphological, and biogeographical data. *Cladistics* 28(3)**,** 271-299.

Korall, P., and Pryer, K.M. (2014). Global biogeography of scaly tree ferns (Cyatheaceae): evidence for Gondwanan vicariance and limited transoceanic dispersal. *J Biogeogr* 41(2)**,** 402-413. doi: 10.1111/jbi.12222.

Labiak, P.H., Sundue, M., Rouhan, G., Hanks, J.G., Mickel, J.T., and Moran, R.C. (2014). Phylogeny and historical biogeography of the lastreopsid ferns (Dryopteridaceae). *American Journal of Botany* 101(7)**,** 1207-1228.

Lavin, M., Herendeen, P.S., and Wojciechowski, M.F. (2005). Evolutionary rates analysis of Leguminosae implicates a rapid diversification of lineages during the tertiary. *Systematic Biology* 54(4)**,** 575-594.

Lehnebach, C.A., Winkworth, R.C., Becker, M., Lockhart, P.J., and Hennion, F. (2017). Around the pole: evolution of sub‐Antarctic *Ranunculus*. *Journal of Biogeography* 44(4)**,** 875-886.

Leslie, A.B., Beaulieu, J.M., Rai, H.S., Crane, P.R., Donoghue, M.J., and Mathews, S. (2012). Hemisphere-scale differences in conifer evolutionary dynamics. *Proceedings of the National Academy of Sciences U.S.A.* 109(40)**,** 16217-16221.

Li, X., Duke, N.C., Yang, Y., Huang, L., Zhu, Y., Zhang, Z., et al. (2016). Re-evaluation of phylogenetic relationships among species of the mangrove genus *Avicennia* from Indo-West Pacific based on multilocus analyses. *PloS One* 11(10)**,** e0164453.

Liu, H., and Schneider, H. (2013). Evidence supporting *Davallia canariensis* as a Late Miocene relict endemic to Macaronesia and Atlantic Europe. *Australian Systematic Botany* 26(5)**,** 378-385.

Marcussen, T., and Meseguer, A.S. (2017). Species-level phylogeny, fruit evolution and diversification history of *Geranium* (Geraniaceae). *Molecular Phylogenetics and Evolution* 110**,** 134-149.

Meseguer, A.S., Aldasoro, J.J., and Sanmartín, I. (2013). Bayesian inference of phylogeny, morphology and range evolution reveals a complex evolutionary history in St. John’s wort (*Hypericum*). *Molecular Phylogenetics and Evolution* 67(2)**,** 379-403.

Meudt, H., M., R.A.B., M., P.J., Evonne, L., J., G.J.P., and C., A.D. (2015a). Is genome downsizing associated with diversification in polyploid lineages of *Veronica*? *Botanical Journal of the Linnean Society* 178(2)**,** 243-266. doi: doi:10.1111/boj.12276.

Meudt, H.M., Lockhart, P.J., and Bryant, D. (2009). Species delimitation and phylogeny of a New Zealand plant species radiation. *BMC Evolutionary Biology* 9(1)**,** 111. doi: 10.1186/1471-2148-9-111.

Meudt, H.M., Prebble, J.M., and Lehnebach, C.A. (2015b). Native New Zealand forget-me-nots (*Myosotis*, Boraginaceae) comprise a Pleistocene species radiation with very low genetic divergence. *Plant Systematics and Evolution* 301(5)**,** 1455-1471.

Mitchell, A., Li, R., Brown, J.W., Schönberger, I., and Wen, J. (2012). Ancient divergence and biogeography of *Raukaua* (Araliaceae) and close relatives in the southern hemisphere. *Australian Systematic Botany* 25(6)**,** 432-446. doi: <https://doi.org/10.1071/SB12020>.

Mitchell, A.D., Heenan, P.B., Murray, B.G., Molloy, B.P.J., and de Lange, P.J. (2009). Evolution of the south-western Pacific genus *Melicytus* (Violaceae): evidence from DNA sequence data, cytology and sex expression. *Australian Systematic Botany* 22(3)**,** 143-157. doi: <https://doi.org/10.1071/SB08042>.

Mummenhoff, K., Linder, P., Friesen, N., Bowman, J.L., Lee, J.Y., and Franzke, A. (2004). Molecular evidence for bicontinental hybridogenous genomic constitution in *Lepidium* sensu stricto (Brassicaceae) species from Australia and New Zealand. *American Journal of Botany* 91(2)**,** 254-261.

Muñoz-Rodríguez, P., Carruthers, T., Wood, J.R., Williams, B.R., Weitemier, K., Kronmiller, B., et al. (2019). A taxonomic monograph of *Ipomoea* integrated across phylogenetic scales. *Nature Plants* 5(11)**,** 1136-1144.

Nargar, K., Molina, S., Wagner, N., Nauheimer, L., Micheneau, C., and Clements, M.A. (2019). Australasian orchid diversification in time and space: molecular phylogenetic insights from the beard orchids (*Calochilus*, Diurideae). *Australian Systematic Botany* 31(6)**,** 389-408.

Nauheimer, L., Schley, R.J., Clements, M.A., Micheneau, C., and Nargar, K. (2018). Australasian orchid biogeography at continental scale: molecular phylogenetic insights from the sun orchids (*Thelymitra*, Orchidaceae). *Molecular Phylogenetics and Evolution* 127**,** 304-319.

Nicolas, A.N., and Plunkett, G.M. (2014). Diversification times and biogeographic patterns in Apiales. *The Botanical Review* 80(1)**,** 30-58.

Nylinder, S., Cronholm, B., de Lange, P.J., Walsh, N., and Anderberg, A.A. (2013). Species tree phylogeny and character evolution in the genus *Centipeda* (Asteraceae): evidence from DNA sequences from coding and non-coding loci from the plastid and nuclear genomes. *Molecular Phylogenetics and Evolution* 68(2)**,** 239-250.

Nylinder, S., Lemey, P., De Bruyn, M., Suchard, M.A., Pfeil, B.E., Walsh, N., et al. (2014). On the biogeography of *Centipeda*: a species-tree diffusion approach. *Systematic Biology* 63(2)**,** 178-191.

Nylinder, S., Swenson, U., Persson, C., Janssens, S.B., and Oxelman, B. (2012). A dated species–tree approach to the trans–Pacific disjunction of the genus *Jovellana* (Calceolariaceae, Lamiales). *Taxon* 61(2)**,** 381-391. doi: <https://doi.org/10.1002/tax.612009>.

Papadopulos, A.S., Baker, W.J., Crayn, D., Butlin, R.K., Kynast, R.G., Hutton, I., et al. (2011). Speciation with gene flow on Lord Howe Island. *Proceedings of the National Academy of Sciences U.S.A.* 108(32)**,** 13188-13193.

Perrie, L., and Brownsey, P. (2007). Molecular evidence for long‐distance dispersal in the New Zealand pteridophyte flora. *Journal of Biogeography* 34(12)**,** 2028-2038.

Pirie, M.D., Humphreys, A.M., Galley, C., Barker, N.P., Verboom, G.A., Orlovich, D., et al. (2008). A novel supermatrix approach improves resolution of phylogenetic relationships in a comprehensive sample of danthonioid grasses. *Molecular Phylogenetics and Evolution* 48(3)**,** 1106-1119.

Pirie, M.D., Lloyd, K.M., Lee, W.G., and Linder, H.P. (2010). Diversification of *Chionochloa* (Poaceae) and biogeography of the New Zealand Southern Alps. *Journal of Biogeography* 37(2)**,** 379-392.

Prebble, J.M., Cupido, C.N., Meudt, H.M., and Garnock-Jones, P.J. (2011). First phylogenetic and biogeographical study of the southern bluebells (*Wahlenbergia*, Campanulaceae). *Molecular Phylogenetics and Evolution* 59(3)**,** 636-648.

Puente-Lelièvre, C., Harrington, M.G., Brown, E.A., Kuzmina, M., and Crayn, D.M. (2013). Cenozoic extinction and recolonization in the New Zealand flora: the case of the fleshy-fruited epacrids (Styphelieae, Styphelioideae, Ericaceae). *Molecular Phylogenetics and Evolution* 66(1)**,** 203-214.

Sancho, G., de Lange, P.J., Donato, M., Barkla, J., and Wagstaff, S.J. (2015). Late Cenozoic diversification of the austral genus *Lagenophora* (Astereae, Asteraceae). *Botanical Journal of the Linnean Society* 177(1)**,** 78-95.

Särkinen, T., Bohs, L., Olmstead, R.G., and Knapp, S. (2013). A phylogenetic framework for evolutionary study of the nightshades (Solanaceae): a dated 1000-tip tree. *BMC Evolutionary Biology* 13(1)**,** 214.

Sauquet, H., Ho, S.Y., Gandolfo, M.A., Jordan, G.J., Wilf, P., Cantrill, D.J., et al. (2012). Testing the impact of calibration on molecular divergence times using a fossil-rich group: the case of *Nothofagus* (Fagales). *Systematic Biology* 61(2)**,** 289-313.

Schuettpelz, E., and Pryer, K.M. (2007). Fern phylogeny inferred from 400 leptosporangiate species and three plastid genes. *Taxon* 56(4)**,** 1037-1050.

Schuster, T.M., Setaro, S.D., and Kron, K.A. (2013). Age estimates for the Buckwheat family Polygonaceae based on sequence data calibrated by fossils and with a focus on the amphi-Pacific *Muehlenbeckia*. *PLOS ONE* 8(4)**,** e61261. doi: 10.1371/journal.pone.0061261.

Schwery, O., Onstein, R.E., Bouchenak‐Khelladi, Y., Xing, Y., Carter, R.J., and Linder, H.P. (2015). As old as the mountains: the radiations of the Ericaceae. *New Phytologist* 207(2)**,** 355-367.

Slovák, M., Kučera, J., Lack, H.W., Ziffer-Berger, J., Melicharková, A., Záveská, E., et al. (2018). Diversification dynamics and transoceanic Eurasian-Australian disjunction in the genus *Picris* (Compositae) induced by the interplay of shifts in intrinsic/extrinsic traits and paleoclimatic oscillations. *Molecular Phylogenetics and Evolution* 119**,** 182-195.

Smissen, R., Garnock-Jones, P., and Chambers, G. (2003). Phylogenetic analysis of ITS sequences suggests a Pliocene origin for the bipolar distribution of *Scleranthus* (Caryophyllaceae). *Australian Systematic Botany* 16(3)**,** 301-315.

Spalik, K., Piwczyński, M., Danderson, C.A., Kurzyna‐Młynik, R., Bone, T.S., and Downie, S.R. (2010). Amphitropic amphiantarctic disjunctions in Apiaceae subfamily Apioideae. *Journal of Biogeography* 37(10)**,** 1977-1994.

Stöckler, K., Daniel, I.L., and Lockhart, P.J. (2002). New Zealand kauri (*Agathis australis* (D. Don) Lindl., Araucariaceae) survives Oligocene drowning. *Systematic Biology* 51(5)**,** 827-832.

Tay, M.L., Meudt, H.M., Garnock-Jones, P.J., and Ritchie, P.A. (2010). DNA sequences from three genomes reveal multiple long-distance dispersals and non-monophyly of sections in Australasian *Plantago* (Plantaginaceae). *Australian Systematic Botany* 23(1)**,** 47-68.

Thomas, N., Bruhl, J.J., Ford, A., and Weston, P.H. (2014). Molecular dating of Winteraceae reveals a complex biogeographical history involving both ancient Gondwanan vicariance and long‐distance dispersal. *Journal of Biogeography* 41(5)**,** 894-904.

Thornhill, A.H., Ho, S.Y., Külheim, C., and Crisp, M.D. (2015). Interpreting the modern distribution of Myrtaceae using a dated molecular phylogeny. *Molecular Phylogenetics and Evolution* 93**,** 29-43.

Vasconcelos, T.N., Proença, C.E., Ahmad, B., Aguilar, D.S., Aguilar, R., Amorim, B.S., et al. (2017). Myrteae phylogeny, calibration, biogeography and diversification patterns: increased understanding in the most species rich tribe of Myrtaceae. *Molecular Phylogenetics and Evolution* 109**,** 113-137.

Verboom, G.A., Linder, H.P., and Stock, W.D. (2003). Phylogenetics of the grass genus *Ehrharta*: evidence for radiation in the summer‐arid zone of the South African Cape. *Evolution* 57(5)**,** 1008-1021.

von Hagen, K.B., and Kadereit, J.W. (2001). The phylogeny of *Gentianella* (Gentianaceae) and its colonization of the southern hemisphere as revealed by nuclear and chloroplast DNA sequence variation. *Organisms Diversity & Evolution* 1(1)**,** 61-79.

von Mering, S. (2013). *Systematics, phylogeny and biogeography of Juncaginaceae.* Ph. D. dissertation, Johannes Gutenberg-Universität Mainz, Mainz, Germany.

von Mering, S., and Kadereit, J.W. (2015). Phylogeny, biogeography and evolution of *Triglochin* L. (Juncaginaceae)–morphological diversification is linked to habitat shifts rather than to genetic diversification. *Molecular Phylogenetics and Evolution* 83**,** 200-212.

Wagstaff, S., and Breitwieser, I. (2002). Phylogenetic relationships of New Zealand Asteraceae inferred from ITS sequences. *Plant Systematics and Evolution* 231(1-4)**,** 203-224.

Wagstaff, S.J., Breitwieser, I., and Ito, M. (2011). Evolution and biogeography of *Pleurophyllum* (Astereae, Asteraceae), a small genus of megaherbs endemic to the subantarctic islands. *American Journal of Botany* 98(1)**,** 62-75.

Wagstaff, S.J., Breitwieser, I., and Swenson, U. (2006). Origin and relationships of the austral genus *Abrotanella* (Asteraceae) inferred from DNA sequences. *Taxon* 55(1)**,** 95-106.

Wagstaff, S.J., and Clarkson, B.R. (2012). Systematics and ecology of the Australasian genus *Empodisma* (Restionaceae) and description of a new species from peatlands in northern New Zealand. *PhytoKeys* (13)**,** 39.

Wagstaff, S.J., Dawson, M.I., Venter, S., Munzinger, J., Crayn, D.M., Steane, D.A., et al. (2010). Origin, diversification, and classification of the Australasian genus *Dracophyllum* (Richeeae, Ericaceae). *Annals of the Missouri Botanical Garden* 97(2)**,** 235-258.

Wagstaff, S.J., Martinsson, K., and Swenson, U. (2000). Divergence estimates of *Tetrachondra hamiltonii* and *T. patagonica* (Tetrachondraceae) and their implications for austral biogeography. *New Zealand Journal of Botany* 38(4)**,** 587-596.

Wagstaff, S.J., and Tate, J.A. (2011). Phylogeny and character evolution in the New Zealand endemic genus *Plagianthus* (Malveae, Malvaceae). *Systematic Botany* 36(2)**,** 405-418.

Wagstaff, S.J., and Wege, J. (2002). Patterns of diversification in New Zealand Stylidiaceae. *American Journal of Botany* 89(5)**,** 865-874.

Xie, L., Wen, J., and Li, L.-Q. (2011). Phylogenetic analyses of *Clematis* (Ranunculaceae) based on sequences of nuclear ribosomal ITS and three plastid regions. *Systematic Botany* 36(4)**,** 907-921.

Zerega, N.J., Clement, W.L., Datwyler, S.L., and Weiblen, G.D. (2005). Biogeography and divergence times in the mulberry family (Moraceae). *Molecular Phylogenetics and Evolution* 37(2)**,** 402-416.

Zhang, L.-B., and Renner, S. (2003). The deepest splits in Chloranthaceae as resolved by chloroplast sequences. *International Journal of Plant Sciences* 164(S5)**,** S383-S392.
